# The Characterization of a Novel Phage, pPa_SNUABM_DT01, Infecting *Pseudomonas aeruginosa*

**DOI:** 10.3390/microorganisms9102040

**Published:** 2021-09-27

**Authors:** Jun Kwon, Sang Wha Kim, Sang Guen Kim, Jeong Woo Kang, Won Joon Jung, Sung Bin Lee, Young Min Lee, Sib Sankar Giri, Cheng Chi, Se Chang Park

**Affiliations:** 1 Laboratory of Aquatic Biomedicine, Research Institute for Veterinary Science, College of Veterinary Medicine, Seoul National University, Seoul 08826, Korea; kjun1002@snu.ac.kr (J.K.); blackcat9201@snu.ac.kr (S.W.K.); imagine0518@snu.ac.kr (S.G.K.); kck90@snu.ac.kr (J.W.K.); cwj0125@snu.ac.kr (W.J.J.); lsbin1129@snu.ac.kr (S.B.L.); mushhama@snu.ac.kr (Y.M.L.); ssgiri@snu.ac.kr (S.S.G.); 2 Laboratory of Aquatic Nutrition and Ecology, College of Animal Science and Technology, Nanjing Agricultural University, Nanjing 210095, China; chicheng@njau.edu.cn

**Keywords:** bacteriophage, taxonomy, *Myoviridae*, *Pseudomonas aeruginosa*

## Abstract

The bacterial genus *Pseudomonas* is a common causative agent of infections in veterinary medicine. In this study, we focused on *Pseudomonas aeruginosa* canine otitis externa isolates. Due to prolonged antibiotic treatment of otitis externa, antibiotic resistance is common and has become a major complication. Many alternatives to antibiotics have been studied, with bacteriophages emerging as the most promising alternatives. Here, we isolated and characterized a novel phage, pPa_SNUABM_DT01, by investigating its morphology, growth, lysis kinetics, and genomic characteristics. Phages have a vigorous capacity to eliminate bacterial cells through bacterial lysis. This capacity is dependent on the multiplicity of infection (MOI), but even at low MOIs, the phage successfully inhibited bacterial regrowth. The phage genome was 265,520 bp in size and comprised 312 putative open reading frames (ORFs). Comparative genome analysis demonstrated that the phage is a novel species in *Myoviridae*. The nucleotide similarity was moderately high compared with the *Pseudomonas* virus, Noxifer. However, a phylogenetic analysis and a dot plot indicated that pPa_SNUABM_DT01 is not closely related to the *Phikzvirus* or *Noxifervirus* genus but, instead, belongs to a novel one. The genome comparisons also indicate that the phage, pPa_SNUABM_DT01, could be a novel genus.

## 1. Introduction

Bacteriophage (phage) is a kingdom of viruses that invades bacterial cells while replicating in them [1]. As bacteriophages have the capability to induce bacterial metabolism disruption and death, they are emerging as alternative treatment modalities for bacteria with antibiotics resistance [1,2,3,4]. Ever since the first discovery of bacteriophages in the 1910s, efforts have been made to use them as therapeutic agents [1,4]. The diverse features of lytic bacteriophages, including their host specificity, exponential growth in numbers at the site of infection, and the capability to overcome bacterial resistance, have made them an attractive alternative to antibiotics [1,2,3,4]. Previous studies in bacteriophage therapy indicate that one of the crucial factors for successful phage therapy is appropriate phage selection. Some biological features, such as phage specificity, efficacy, and lytic phage, should be considered in order to select suitable phages [5,6].

Jumbo-phages are the phages with genomes larger than 200 kbp [7]. As jumbo-phage genomes are large, they are highly diverse and possess more genes than phages with short genomes [7]. The large genome is the result of evolution and adaptation [7]. Therefore, the study of this genome is important for understanding phage infection mechanisms and life cycles, although a number of genes remained uncharacterized [7].

*Pseudomonas aeruginosa* is an encapsulated, Gram-negative, and rod-shaped bacterium that is one of the most commonly isolated species from canine otitis [8,9]. As canine otitis externa itself is a common disease, as 10–20% of dogs that have visited veterinarians have been confirmed to have occurrence, *P. aeruginosa* also becomes one of the bacterial species to be noted in dogs [10]. *P. aeruginosa* commonly shows intrinsic resistance to various antibiotics, resulting in a number of pan-resistant isolates [11]. This is attributable to various factors, including mutational changes in genes constituting multidrug efflux systems and horizontal gene transfer, making their treatment options very limited [11].

In this study, we isolated and characterized bacteriophage pPa_SNUABM_DT01 infecting *P. aeruginosa* clinical isolate. The morphology, biological behaviors, and genomic features of the phage were examined and described. A comparative genome analysis suggested that this phage is a novel genus and species within *Myoviridae*. As the phage can lyse a *P. aeruginosa* multidrug resistant isolate from a canine otitis externa case in the Republic of Korea, the phage has the potential to become one of the good therapeutic options.

## 2. Materials and Methods

### 2.1. Bacterial Strain and Growth Conditions

*P. aeruginosa* strains were isolated from canines with otitis externa at a local animal hospital in the Republic of Korea. Bacterial swab samples were collected from the diseased dog ear canals and kept in a transport medium at 4 °C during shipping. Samples were then spread on tryptic soy agar (TSA; Difco) and incubated overnight at 37 °C. Dominant colonies were subcultured under the same conditions for three consecutive generations. The 16S rDNA genes were purified from the isolates and were sequenced. Sequence identification was performed using the basic local alignment search tool (BLAST) (Macrogen Korea, Seoul, Korea) [12]. These bacterial strains were then stored in tryptic soy broth (TSB; Difco, USA) containing 15% glycerol at −70 °C until further use.

### 2.2. Isolation and Characterization of Bacteriophage

Isolation of the new phage strain was conducted as previously described [13]. Water samples were collected from diverse environments in Chungju-city, Chungcheongbuk-do, Republic of Korea, and were filtered through a filter with a 0.45 um pore size (Merck Millipore, Billerica, MA, USA) and mixed (1%, *v*/*v*) with an overnight culture of *P. aeruginosa* in TSB (Difco). After 24 h of incubation at 37 °C, a conventional double-layer agar plate assay was conducted for phage plaque detection. One plaque was selected and streaked out for subculture, and this was repeated five times to obtain a pure phage isolate. This phage was designated as pPa_SNUABM_DT01.

The characterization of pPa_SNUABM_DT01 was conducted as previously described [13]. An adsorption assay was performed, followed by a one-step growth assay for the latency period and burst-size determination. To investigate adsorption kinetics, the host bacteria cultured in TSB and bacterial cells in the exponential phase were infected with phage lysate, i.e., final MOI 0.01. The mixture was incubated at 37 °C, and 100 μL aliquots were collected at 0, 0.5, 1, 2, 3, 5, 7, 10, 15, 20, and 30 min after inoculation. To determine the unadsorbed phage concentration, the aliquots were added to 900 μL of SM buffer and centrifuged at 13,000× *g* for 3 min. The supernatant was collected for the double-layer plaque assay.

For the one-step growth assay (done in triplicate), the host strain was cultured in TSB to reach 1.5 × 10^6^ CFU/mL in the exponential phase. Phage lysate was added to reach MOI = 0.01, followed by a 5 min adsorption period. After 2 min of centrifugation at 14,000× *g*, the supernatant was removed, and an identical volume of TSB (37 °C) was added. After resuspension, 100 μL of bacteria and phage suspension was sampled in 10-min intervals for 140 min and diluted with PBS for the double-layer plaque assay.

### 2.3. Transmission Electron Microscopy (TEM) of pPa_SNUABM_DT01

The phage, pPa_SNUABM_DT01, was concentrated via polyethylene glycol precipitation in a sodium chloride-magnesium sulfate (SM) buffer (50 mM Tris at pH 7.5, 100 mM NaCl, and 10 mM MgSO_4_). Phages attached to the grid were negatively stained with 2% uranyl acetate for 1 min. After air-drying for 10 min, TEM (Talos L120C, FEI, Hillsboro, OR, USA) was performed at 120 kV. The average size of the head and contractile tail was measured using five randomly chosen phages from the TEM scans.

### 2.4. In Vitro Planktonic Bacterial Lysis Assay

This assay determined the lytic efficacy of pPa_SNUABM_DT01 using bacteria-phage suspensions with MOI = 0.01, 0.1, 1, 10 and 100, as well as a no-phage control. Suspensions were mixed by adding phage lysate to the exponential phase host strain which reached a 0.05 OD value (1 × 10^7^ CFU/mL), and then cultured in a shaking incubator at 37 °C (150 rpm). The optical density at 600 nm (OD_600_) was measured at 0, 1, 2, 3, 4, 5, 6, 8, 10, and 12 h. The entire process was repeated six consecutive times.

### 2.5. Complete Genome Sequencing and Annotation

The genomic DNA of pPa_SNUABM_DT01 was extracted following previously published procedures [13]. The complete genome was sequenced by Macrogen Korea (Seoul, Korea), using the Illumina Hiseq2500 platform (San Diego, CA, USA).

Open reading frames (ORFs) of the genome were predicted and validated for putative protein function identification using Rapid Annotation in Subsystem Technology v2.0 (RAST), GeneMarkS v4.28, and the BLAST from NCBI (including BLASTp, BLASTX, PSI-BLAST, and HHpred) [14,15,16,17]. Transfer RNA was detected using tRNAscan-SE v.2.0 [18]. The genomic data were manually annotated using Unipro UGENE v35.0.

### 2.6. Comparative Genome Analysis

Genome sequences were obtained from the GenBank database. The whole genome phylogeny was constructed using the Genome-BLAST Distance Phylogeny method in the Virus Classification and Tree Building Online Resource (VICTOR) [19]. The resulting intergenomic distances (100 replicates each) were used to infer a balanced minimum evolution tree with branch support via FASTME, including subtree pruning and regrafting postprocessing for D0. The tree was visualized using FigTree [20,21]. For conserved gene phylogeny construction, we utilized major capsid protein and terminase large subunit genes and used MEGA v10.1.8 software with the maximum-likelihood method with 1000 bootstrap replications [22]. A dot plot was constructed using the default settings in the Gepard application [23]. For the ANI calculation, a Kostas lab ANI calculator (Kostas lab, Atlanta, GA, USA) was used. With Mauve, the genomes of the *Pseudomonas* phages, pPa_SNUABM_DT01, phiKZ, and Noxifer, were compared [24].

## 3. Results

### 3.1. Isolation and Biological Characterization of pPa_SNUABM_DT01

**Figure 1:**
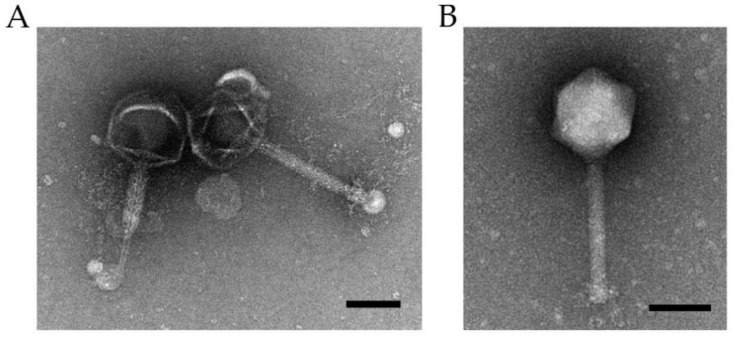
Transmission electron micrograph of pPa_SNUABM_DT01. (**A**) Contracted phage showing tail tube (left) and extended phage (right). (**B**) Extended phage showing clear icosahedral head morphology. Scale bars = 100 nm.

The *Pseudomonas* phage was isolated from water samples from Chungju-city, Chungcheongbuk-do, Korea. Morphological analysis was performed by TEM, based on the previous study [25]. The isolated phage, pPa_SNUABM_DT01, was classified morphologically as of the *Myoviridae* family (Figure 1). The phage has an icosahedral head, a 136.2 ± 2.3 nm diameter, and a tail 224.5 ± 7.2 nm in length.

**Figure 2:**
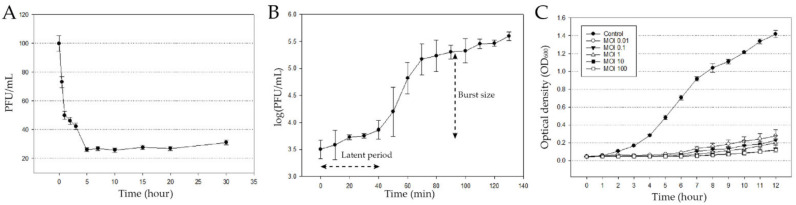
Biological features of pPa_SNUABM_DT01. (**A**) Adsorption assay of pPa_SNUABM_DT01 to host bacterial strain. (**B**) One-step growth curve of pPa_SNUABM_DT01 to host bacterial strain. (**C**) In vitro planktonic cell lysis assay of pPa_SNUABM_DT01 at different MOIs against host bacteria.

The phage infection kinetics were investigated (Figure 2). 74% of the phage was adsorbed within 5 min. The adsorption constant k was calculated as mentioned in Hyman et al. (2009), k = 1.79 × 10^−6^ mL/min [26]. A one-step growth assay showed that the burst size per virion is 10^2.17^, and the latent period is 40 min (Figure 2B).

Cell lysis capacity against planktonic bacterial cells was determined (Figure 2C). The phage-treated groups showed significant bacterial growth inhibition, compared to the control group. The OD_600_ value showed that the cell lysis capacity of the phage is dependent on the MOI. Moreover, even a low MOI is enough to inhibit bacterial regrowth.

Highly virulent phage has a big advantage in phage therapy. Bacterial regrowth is not a rare case [27]. Although it does not always mean phage resistance, it can induce coevolution in phage-bacteria communities, and phage resistance in the end [28,29]. Therefore, previous studies take the cocktail method as a solution, and these cocktails contain not only different phages, but also antibiotics or other synergetic substances [30,31,32,33,34].

### 3.2. Genome Analysis of pPa_SNUABM_DT01

#### 3.2.1. General Features of the Phage Genome

**Figure 3:**
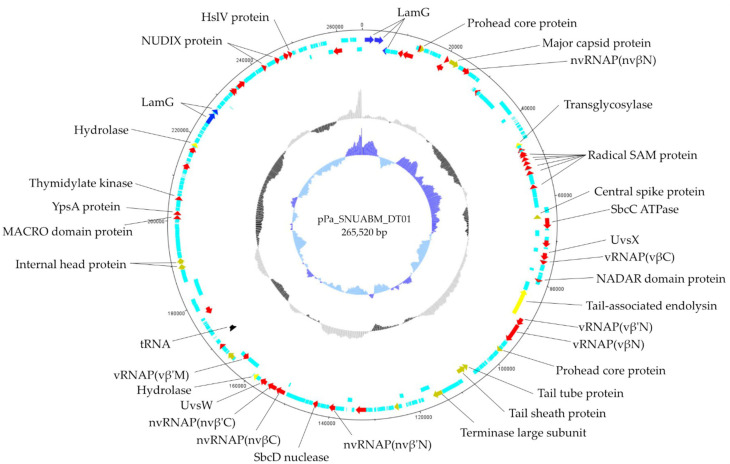
Genome map of pPa_SNUABM_DT01. The functional ORFs are indicated by specific colors according to their functional categories. The GC plot is indicated by the black and grey hologram, and the GC skew is demonstrated with the blue hologram. Red: nucleotide-metabolism-related ORFs; Yellow: lysis related ORFs; Green: structure-and-packaging-related ORFs. Black: tRNA; Sky-blue: hypothetical ORFs.

The phage genome was a 265,520 bp long circular double-stranded DNA, with a GC content of 56.0% (GenBank accession number: MW735835) (Figure 3). We predicted 312 putative open reading frames (ORFs), with 253 genes (81.09%) located on the sense strand, and 59 genes (18.91%) on the antisense strand. The functional predictions of the ORFs were performed through a protein sequence homology search. The results classified 66 (21.15%) ORFs as functional and 149 as hypothetical proteins.

The ORF functions were categorized into four sections: structural and packaging (e.g., prohead core gene, major capsid protein, central spike protein, baseplate hub assembly protein, internal head proteins), nucleotide metabolism (e.g., RNAP genes, DNA polymerase, thymidylate kinase, endonuclease, SbcCD complex), lysis (e.g., transglycosylase, peptidoglycan hydrolase), and other functions (e.g., Laminin G, tyrosine-protein phosphatase) (Supplementary Table S1). The phage genome encodes for two tRNA-specific amino acids, which included asparagine and aspartic acid.

#### 3.2.2. Specific Features of the Phage Genome

The searching for RNA polymerase (RNAP) genes was performed by BLAST and HHpred searching tools. Gp082, 092, 093, 148, 160, 161, and 168 were predicted as RNAPs. These gene were assigned to each subunit through an amino acid sequence-based homology search. We identified virion-associated RNAP (vRNAP) and nonvirion-associated RNAP (nvRNAP) subunits. Four genes (gp082, 092, 093, and 168) were homologous to vRNAP and assigned to vRNAP subunits: gp093 to vβN, gp082 to vβC, gp092 to vβN, and gp168 to vβ′M. The same assignments were made for the nvRNAP subunit genes: gp025 to nvβN, gp160 to nvβC, gp148 to nvβ′N, and gp161 to nvβ′C. One of the RNAP subunit genes (vβ′C) was considered to be comprised of hypothetical genes. The presence of vRNAP and nvRNAP suggests that phage pPa_SNUABM_DT01 has a similar transcriptional strategy as phiKZ [35,36]. Furthermore, the amino acid sequence similarity search showed that the phage RNAP genes were homologous to phiKZ RNAP genes [35,36]. Hence, the novel phage has a relatively close evolutionary relationship with phiKZ but diverged earlier on.

YspA gene protein (a member of the SLOG superfamily), a NUDIX domain, a NADAR domain, and a MACRO domain were found by homology searching, i.e., gp213 to YspA, gp283 and gp286 to the NUDIX domain, gp088 to the NADAR domain, gp212 to the MACRO domain. YspA protein, by fusing to each domain, is known to be involved in NAD metabolism and processing ADP-ribose derivatives in bacteria [37,38]. They are also considered to be concerned in bacterial RNA-dependent RNA polymerase modules [37].

The thymidylate kinase, gp215, is an enzyme that catalyzes DNA precursor synthesis. This enzyme has a key role in pyrimidine synthesis, so it supports phage proliferation. This enzyme allows the phage to be less reliant on host enzymes [39].

Several DNA repair genes were recognized. The SbcCD complex was recognized at: gp072; SbcC and gp153; and SbcD, separated into two subunits. The SbcC subunit is ATPase and SbcD is a DNA exonuclease. The complex is involved in DNA repair, and so it has effects on phage stability and proliferation [40]. UvsX recombinase and UvsW ATP-dependent helicase genes were recognized at: gp080; UvsX, and gp163; and UvsW. These proteins also coordinate for DNA repair [41]. Six radical S-adenosyl-L-methionine (SAM) genes were found at gp057, 058, 059, 060, 062, and 066. Radical SAM proteins are associated with DNA repair and host-metabolism boosting during infection [42].

The internal head proteins were predicted to be gp200 and 201. This protein is a cylindrical structure that has been found in some phiKZ-like jumbo-phages, e.g., 201phi-1, phiPA3, phiEL, and OBP [43]. Previous studies have shown that this protein is essential to DNA ejection and phage morphogenesis [44]. However, no comprehensive description exists of the protein’s exact activities [45].

Using a conserved domain search, we found several laminin G (LamG) domains in gp001, 002, 003, 256, and 258. Its function is unclear, but McCutcheon et al. predicted that this domain acts as a receptor-binding protein during host recognition [46]. Similarly, Fraser et al. suggested that Ig-like domains on bacteriophages may play an accessory role in phage-bacterial cell surface interactions [46]. To verify the relationship between LamG and Ig-like domains, we used a PSI-BLAST search. The results reveal that LamG domains in the phage genome have significant sequence similarity to several Ig-like domains. This supports the theory that LamG domains may have similar functions to Ig-like domains in phage-bacterial cell surface interactions.

### 3.3. Comparative Analysis of the Phage Genome

A nucleotide sequence similarity search showed that the *Pseudomonas* virus, Noxifer, has relatedness to pPa_SNUABM_DT01 (83.5% similarity). To investigate the genetic distance between the two phages, a two-way ANI was calculated with whole genome sequences. The ANI value of pPa_SNUABM_DT01 and Noxifer were moderately high, at 74.7%. However, only five hits were detected, which is insufficient to estimate the two-way ANI value.

**Figure 4:**
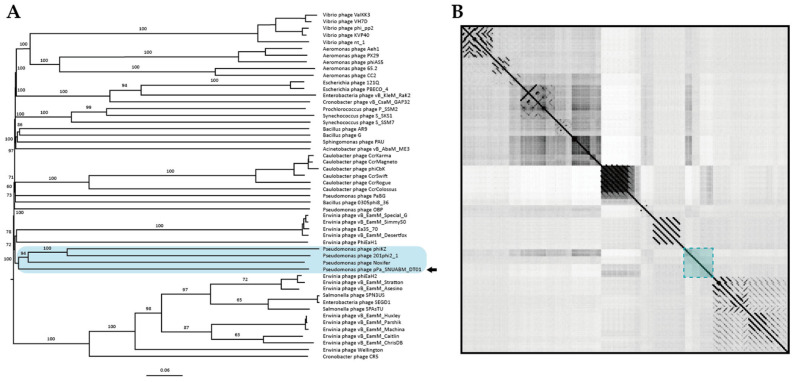
Whole genome comparison of the pPa_SNUABM_DT01 genome and jumbo-phages. Skyblue box: the cluster including pPa_SNUABM_DT01. (**A**) Phylogenetic tree constructed using VICTOR with genome sequences of jumbo-phages. (**B**) Dot plot constructed with concatenated genome sequences used in phylogeny.

To investigate phage taxonomy, a phylogeny tree and a dot plot were constructed with jumbo-bacteriophage genome sequences belonging to various genera (Figure 4). The phylogeny revealed that the phage, pPa_SNUABM_DT01, was included in a cluster with Noxifer and phiKZ (Skyblue box, Figure 4A). According to the reconstructed phylogeny, Noxifer (genus *Noxifervirus*) turned out to be the closest virus to pPa_SNUABM_DT01. However, the evolutionary distance between the two viruses was not close. This was also shown by a genome comparison with a dot plot, as they demonstrate that pPa_SNUABM_DT01 has less genetic similarity to other phages (Figure 4B). To confirm the taxon of the phage, a more detailed phylogeny was reconstructed with the viral genomes of *Pseudomonas* bacteriophages belonging to *Myoviridae* (Supplementary Figure S1).

**Figure 5:**
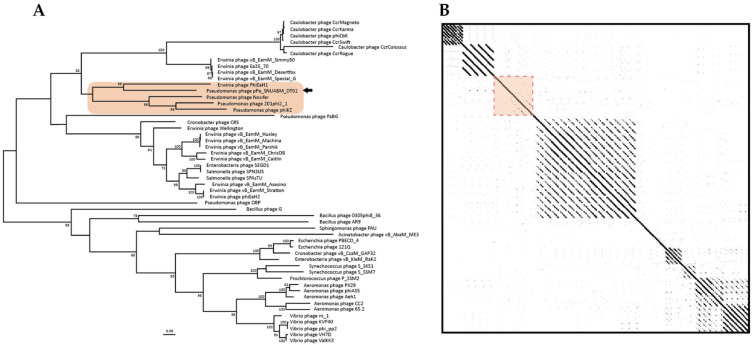
Conserved gene (major capsid protein) comparison of pPa_SNUABM_DT01 and jumbo-phages. Orange box: the cluster including pPa_SNUABM_DT01. (**A**) Phylogenetic tree constructed using MEGA-X by maximum-likelihood with major capsid protein sequences of pPa_SNUABM_DT01 and jumbo-phages. (**B**) Dot plot constructed with concatenated genome sequences used in phylogeny at a word size of 12.

**Figure 6:**
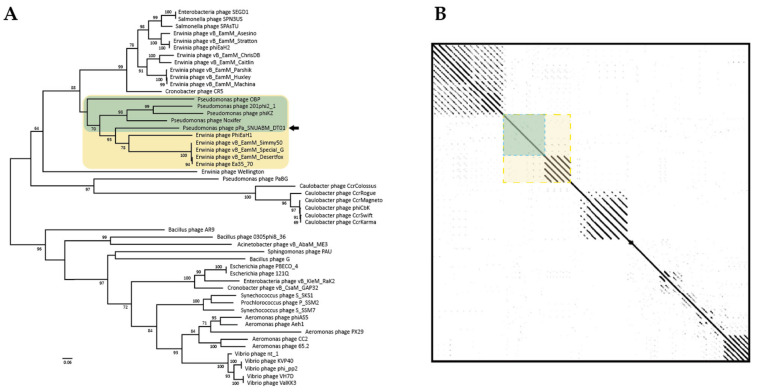
Conserved gene (terminase large subunit) comparison of pPa_SNUABM_DT01 and jumbo-phages. Yellow box: the cluster including pPa_SNUABM_DT01. Green box: the phages do not show diagonal lines in the cluster (Yellow box). (**A**) Phylogenetic tree constructed using MEGA-X by maximum-likelihood with major capsid protein sequences of pPa_SNUABM_DT01 and jumbo-phages. (**B**) Dot plot constructed with concatenated genome sequences used in phylogeny at a word size of 12.

We used a conserved gene phylogeny to confirm whether the phage, pPa_SNUABM_DT01, is a novel species or a further genus (Figure 5 and Figure 6). Two conserved genes (major capsid protein and terminase large subunit) of the jumbo-phages were used for phylogeny construction. As shown in the two phylogenetic trees, pPa_SNUABM_DT01 was close to phage Noxifer in the distance of branches but were not involved in the small cluster containing Noxifer. The phage, pPa_SNUABM_DT01, was separated and evolved long ago.

## 4. Conclusions

Antimicrobial resistance is one of the major concerns of the medical sciences [8,9,10,11]. The greater this concern is, the more research into the alternative measures [1,2,3,4,13]. Bacteriophage is the most promising alternative for antibiotics. Here, we isolated a bacteriophage, pPa_SNUABM_DT01, infecting *P. aeruginosa*. The phage showed high virulence to planktonic cells. The phage virulence used to be dependent on the MOI, but in the case of this phage, bacterial regrowth was inhibited even at a low MOI. As the phage efficacy to eliminate bacteria is one of the most important factors in phage therapy, this phage is a potential candidate for administration.

Several genetic features were revealed by the genome analysis. The phage genome possesses a 265,520 bp size, which is within the common genome size range of jumbo-phages [7]. A total of 312 putative ORFs were recognized and, of them, 63 ORFs were predicted functional. Two RNA genes were recognized. Seven putative RNAP subunit genes were found. By homology searching, four vRNAP and three nvRNAP subunits were predicted. The unpredicted RNAP subunit genes were considered to be comprised of hypothetical ORFs. A total of five LamG genes were found. Because these genes relate to Ig-like domains based on the PSI-BLAST search, we predict their functions may be similar, i.e., they play an accessory role in phage-bacterial cell surface interaction [7,46].

The evidence that the phage is a novel species is shown in the genome comparison. A whole genome BLASTn search, and an ANI calculation, revealed that the phage genome is most similar to the *Pseudomonas* phage, Noxifer. However, the genome comparison using phylogeny and a dot plot showed that the phage, pPa_SNUABM_DT01, was separated long ago from the closest species, Noxifer. The results also suggest that the phage, pPa_SNUABM_DT01, could be a novel genus.

## Data Availability

The datasets presented in this study can be found in online repositories. The genome has been submitted to NCBI and can be found under the GenBank accession number MW735835.

## Supplementary Materials

The following are available online at https://www.mdpi.com/article/10.3390/microorganisms9102040/s1, Table S1: Categories and homologies of the putative functional ORFs in pPa_SNUABM_DT01; Figure S1: Whole genome comparative analysis of pPa_SNUABM_DT01, and *Myoviridae* phages.
