# Supplementary material for: The Characterization of a Novel Phage, pPa_SNUABM_DT01, Infecting Pseudomonas aeruginosa
Source: Microorganisms. 2021 Sep 27;9(10):2040. doi: 10.3390/microorganisms9102040 (PMC8541153; doi:10.3390/microorganisms9102040)
Supplement: Supplementary file 1 [file microorganisms-09-02040-s001.zip › microorganisms-1362964-supplementary.pdf]

Supplementary Table S1. Categories and homologies of the putative functional ORFs in pPa_SNUABM_DT01.

Functional group	Locus tag	Encoded protein [Conserved domain]	Related organism	BLASTp e-value
DNA replication and nucleotide metabolism protein	PA10_00008	putative ribonucleoside-diphosphate reductase subunit beta [Ribonucleotide Reductase, R2/beta subunit, ferritin-like diiron-binding domain (cd01049); NCBI_Conserved_Domain_v3.18]	Pseudomonas phage Noxifer	0
	PA10_00009	putative ribonucleotide reductase subunit beta [Ribonucleotide reductase, alpha subunit (KOG1112); COG_KOG_v1.0]	Candidimonas sp.	0
	PA10_00012	putative deoxycytidine triphosphate deaminase [DCD; 2'-deoxycytidine 5'-triphosphate deaminase (DCD) (PF06559.12); Pfam-A_v33.1]	Pseudomonas phage Noxifer	7E-69
	PA10_00021	putative helicase [DnaB-like helicase C terminal domain (PF03796.16); Pfam-A_v33.1; putative DnaB helicase]	Erwinia phage vB_EamM_RAY	2.00E-81
	PA10_00025	putative non-virion-associated RNA polymerase subunit beta/beta' [putative nvRNAP similar to phiKZ123 protein]	Ralstonia phage RSF1	2.00E-84
	PA10_00030	putative crossover junction endodeoxyribonuclease [RuvC; Crossover junction endodeoxyribonuclease RuvC(PF02075.18); Pfam-A_v33.1]	Vibrio phage pVa-21	2E-13
	PA10_00054	putative 2OG-Fe(II) oxygenase [2OG-Fe(II) oxygenase (PF13759.7); Pfam-A_v33.1]	Ralstonia phage RSF1	1E-15
	PA10_00056	hypothetical protein [Putative 2OG-Fe(II) oxygenase (PF13759.7); Pfam-A_v33.1]	Psychrobacter sp. PRwf-1	3.30E-02
	PA10_00057	radical SAM protein [Radical_SAM; Radical_SAM. Radical SAM superfamily (cd17853); NCBI_Conserved_Domain_v3.18]	Dysosmobacter sp. BX15	1.00E-60
	PA10_00058	radical SAM protein [Radical_SAM; Radical_SAM. Radical SAM superfamily (cd17853); NCBI_Conserved_Domain_v3.18]	Dysosmobacter sp. BX15	2.00E-160
	PA10_00059	radical SAM protein [Radical_SAM; Radical_SAM. Radical SAM superfamily (cd17853); NCBI_Conserved_Domain_v3.18]	Ralstonia phage RSF1	3.00E-75
	PA10_00060	radical SAM protein [Radical_SAM; Radical_SAM. Radical SAM superfamily (cd17853); NCBI_Conserved_Domain_v3.18]	Ralstonia phage RSF1	1.00E-149

	Radical_SAM; Radical_SAM. Radical SAM superfamily (cd17853); NCBI_Conserved_Domain_v3.18		
PA10_00062	radical SAM protein [Radical_SAM; Radical_SAM. Radical SAM superfamily (cd17853); NCBI_Conserved_Domain_v3.18]	Ralstonia phage RSF1	1.00E-33
PA10_00066	radical SAM protein [Radical_SAM; Radical_SAM. Radical SAM superfamily (cd17853); NCBI_Conserved_Domain_v3.18]	Agrobacterium phage Atu_ph07	5E-77
PA10_00072	putative SbcC ATPase [exonuclease SbcC (TIGR00618); TIGRFAMs_v15.0]	Ralstonia phage RP12	3.00E-83
PA10_00077	putative ribonuclease H [rnhA; ribonuclease H (PRK00203); PRK_v6.9]	Xanthomonas phage Xoo-sp14	8.00E-84
PA10_00080	putative UvsX protein [UVSX_BPT4 Recombination and repair protein OS=Enterobacteria phage T4 OX=10665 GN=UVSX PE=1 SV=2 (P04529); Uniprot-SwissProt-viral70_23_Aug_2020]	Ralstonia phage RP12	2E-105
PA10_00082	putative virion-associated RNA polymerase subunit beta/beta' [DNA-directed RNA polymerase subunit B' (PRK07225); PRK_v6.9, putative vRNAP similar to phiKZ149 protein]	Pseudomonas virus phiKZ	4.00E-46
PA10_00092	putative virion-associated RNA polymerase subunit beta/beta' [DNA-directed RNA polymerase subunit beta' (PRK14906); PRK_v6.9, putative vRNAP similar to phiKZ180 protein]	Pseudomonas virus phiKZ	2E-102
PA10_00093	putative virion-associated RNA polymerase subunit beta/beta' [rpoB; DNA-directed RNA polymerase subunit beta (PRK00405); PRK_v6.9, putative vRNAP similar to phiKZ178 protein]	Pseudomonas virus phiKZ	0
PA10_00143	putative DNA polymerase [DNA_pol_B; DNA polymerase family B (PF00136.22); Pfam-A_v33.1]	Ralstonia phage RP31	7e-164
PA10_00148	putative non-virion-associated RNA polymerase subunit beta/beta' [PRK09603; bifunctional DNA-directed RNA polymerase subunit beta/beta'; Reviewed (PRK09603); PRK_v6.9, putative nvRNAP similar to phiKZ055 protein]	Pseudomonas virus phiKZ	1E-60
PA10_00153	putative nuclease SbcCD, subunit D [DNA repair exonuclease SbcCD nuclease subunit (COG0420); COG_KOG_v1.0]	Ralstonia phage RP31	9e-74

	PA10_00160	putative DNA-directed RNA polymerase beta/beta' [PRK14844; bifunctional DNA-directed RNA polymerase subunit beta/beta'; Provisional (PRK14844); PRK_v6.9, putative nvRNAP similar to phiKZ071 protein]	Pseudomonas virus phiKZ	2E-49
	PA10_00161	putative DNA-directed RNA polymerase subunit beta/beta' [rpoC2; DNA-directed RNA polymerase subunit beta'; Provisional (PRK02597); PRK_v6.9, putative nvRNAP similar to phiKZ074 protein]	Pseudomonas virus phiKZ	2E-69
	PA10_00163	putative helicase [UVSW_BPT4 ATP-dependent DNA helicase uvsW OS=Enterobacteria phage T4 OX=10665 GN=uvsW PE=1 SV=2 (P20703); Uniprot-SwissProt-viral70_23_Aug_2020]	Xanthomonas phage Xoo-sp14	2.00E-92
	PA10_00168	putative DNA-directed RNA polymerase subunit beta/beta' [DNA-directed RNA polymerase subunit beta'; Provisional (PRK00566); PRK_v6.9, putative vRNAP similar to phiKZ080 protein]	Pseudomonas virus phiKZ	4.00E-53
	PA10_00176	putative protein tyrosine phosphatase [PTPH_AMEPV Putative tyrosine-protein phosphatase AMV078 OS=Amsacta moorei entomopoxvirus OX=28321 GN=AMV078 PE=3 SV=1 (Q9EMX1); Uniprot-SwissProt-viral70_23_Aug_2020]	-	-
	PA10_00191	putative DNA polymerase [DPOL_BPR69 DNA-directed DNA polymerase OS=Escherichia phage RB69 OX=12353 GN=43 PE=1 SV=1 (Q38087); Uniprot-SwissProt-viral70_23_Aug_2020]	Xanthomonas phage Xoo-sp14	1.00E-134
	PA10_00213	hypothetical protein [YpsA; YspA SLOG family (PF06908.12) domain protein; Pfam-A_v33.1]	Pantoea phage vB_PagS_AAS21	6.00E-15
	PA10_00215	putative thymidylate kinase [thymidylate kinase (PRK13973); PRK_v6.9]	Acidaminococcus timonensis	2.00E-45
	PA10_00220	putative NHN endonuclease [putative NHN endonuclease (PHA00280); PRK_v6.9]		
	PA10_00235	putative thymidylate synthase [TYSY_BPT4 Thymidylate synthase OS=Enterobacteria phage T4 OX=10665 GN=TD PE=1 SV=1 (P00471); Uniprot-SwissProt-viral70_23_Aug_2020]	Pseudomonas phage Phabio	2E-63
	PA10_00241	putative multifunctional tRNA nucleotidyl transferase	Pseudomonas nitritireducens	6.00E-169

		[cca; multifunctional tRNA nucleotidyl transferase/2'3'-cyclic phosphodiesterase/2'nucleotidase/phosphatase; Reviewed (PRK10885); PRK_v6.9]		
	PA10_00270	putative homing endonuclease [GIY-YIG nuclease superfamily protein (PRK00329); PRK_v6.9]	Pseudomonas phage Psa21	3E-13
	PA10_00271	putative ClpXP protease specificity-enhancing factor [ClpXP protease specificity-enhancing factor (PRK11798); PRK_v6.9]	Hafnia alvei	0.00000002
	PA10_00273	putative DNA ligase [DNA_ligase_aden; NAD-dependent DNA ligase adenylation domain (PF01653.19); Pfam-A_v33.1]	Melittangium boletus	7E-122
	PA10_00276	putative dihydrofolate reductase [DHFR_1; Dihydrofolate reductase (PF00186.20); Pfam-A_v33.1]	Sporosarcina sp. HYO08	1E-22
	PA10_00283	putative nudix hydrolase [PLN02325; nudix hydrolase]	Thalassomonas viridans	2.00E-49
	PA10_00286	hypothetical protein [Nudix_Hydrolase; uncharacterized Nudix hydrolase subfamily (cd18881) protein; NCBI_Conserved_Domains_v3.18]	-	-
	PA10_00288	putative ATPase [AAA domain (cd00009) containing protein]	Erwinia phage pEa_SNUABM_50	1.00E-19
	PA10_00290	putative protease [protease_HslV (cd01913) protein; NCBI_Conserved_Domains_v3.18; putative protease HslV]	Bdellovibrionaceae bacterium	3E-16
	PA10_00307	putative ATP-dependent helicase [ATP-dependent helicase HepA (PRK04914); PRK_v6.9]	Ralstonia phage RP12	6.00E-170
structural and packaging protein	PA10_00013	putative prohead core protein protease [Peptidase_S77; Prohead core protein serine protease (PF03420.14); Pfam-A_v33.1]	-	-
	PA10_00023	putative major capsid protein	Xanthomonas phage Xoo-sp14	2.00E-107
	PA10_00071	putative central spike protein [BP45_BPMU Baseplate puncturing device gp45 OS=Escherichia phage Mu OX=10677 GN=Mup45 PE=1 SV=1 (Q9T1V4); Uniprot-SwissProt-viral70_23_Aug_2020]	Salmonella phage vB_SalM_SA002	1E-38
	PA10_00097	putative prohead core protein protease [Peptidase_S77; Prohead core protein serine protease (PF03420.14); Pfam-A_v33.1]	Ralstonia phage RP31	2.00E-49

	PA10_00108	putative tail tube protein [BP54_BPT4 Baseplate tail-tube junction protein gp54OS=Enterobacteria phage T4 OX=10665 GN=54 PE=1 SV=1 (P13341); Uniprot-SwissProt-viral70_23_Aug_2020]	Xanthomonas phage Xoo-sp14	2.00E-68
	PA10_00109	putative tail sheath protein [TSP_BPT4 Tail sheath protein OS=Enterobacteria phage T4 OX=10665 GN=18 PE=1 SV=5 (P13332); Uniprot-SwissProt-viral70_23_Aug_2020]	Ralstonia phage RP12	4.00E-115
	PA10_00113	putative terminase large subunit [Terminase_1; Phage Terminase (PF03354.16); Pfam-A_v33.1]	Xanthomonas phage Xoo-sp14	3.00E-180
	PA10_00128	hypothetical protein [PHUZ_BPDPK Phage tubulin-like protein OS=Pseudomonas phage phiKZ OX=169683 GN=PHIKZ039 PE=1 SV=1 (Q8SDC3); Uniprot-SwissProt-viral70_23_Aug_2020]	Erwinia phage PhiEaH1	3E-20
	PA10_00171	putative baseplate hub assembly protein [GP26_BPT4 Baseplate hub assembly protein gp26 OS=Enterobacteria phage T4 OX=10665 GN=26 PE=1 SV=2 (P13335); Uniprot-SwissProt-viral70_23_Aug_2020]	Bacillus phage vB_BspM_AgentSmith	3E-33
	PA10_00200	putative internal head protein [phiKZ_IP; phiKZ-like phage internal head proteins (PF12699.8); Pfam-A_v33.1]	-	-
	PA10_00201	putative internal head protein [phiKZ_IP; phiKZ-like phage internal head proteins (PF12699.8); Pfam-A_v33.1]	-	-
lysis related protein	PA10_00052	putative transglycosylase	Erwinia phage PhiEaH1	6E-76
	PA10_00091	putative tail-associated endolysin [EXLYS_BPDPK Peptidoglycan hydrolase gp181 OS=Pseudomonas phage phiKZ OX=169683 PE=1 SV=1 (Q8SCY1); Uniprot-SwissProt-viral70_23_Aug_2020]	-	-
	PA10_00165	putative hydrolase [putative hydrolase (PRK10119); PRK_v6.9]	Erwinia phage pEa_SNUABM_43	2E-21
	PA10_00243	putative hydrolase [putative hydrolase (PRK10119); PRK_v6.9]	Pseudomonas phage PaBG	5E-21
Additional functional protein	PA10_00001	putative concanavalin A-like lectin/glucanases superfamily protein [Laminin_G_3 (PF13385); Pfam-A_v33.1]	Proteobacteria bacterium	1E-27
	PA10_00002	putative concanavalin A-like lectin/glucanases superfamily protein	Proteobacteria bacterium	1E-22

		[Laminin_G_3 (PF13385); Pfam-A_v33.1]		
	PA10_00003	putative concanavalin A-like lectin/glucanases superfamily protein [Laminin_G_3 (PF13385); Pfam-A_v33.1]	Candidatus Taylorbacteria bacterium	0.000000002
	PA10_00254	hypothetical protein [PyocinActivator; Pyocin activator protein PrtN (PF11112.9) containing protain; Pfam-A_v33.1]	-	-
	PA10_00256	hypothetical protein [Laminin_G_3 domain (PF13385) containing protein]	-	-
	PA10_00258	hypothetical protein [Laminin_G_3 domain (PF13385) containing protein]	-	-
tRNA	PA10_00179	tRNA-Asn	-	-
	PA10_00180	tRNA-Asp	-	-

Supplementary Figure S1. Whole genome comparative analysis of pPa_SNUABM_DT01, and *Myoviridae* phages

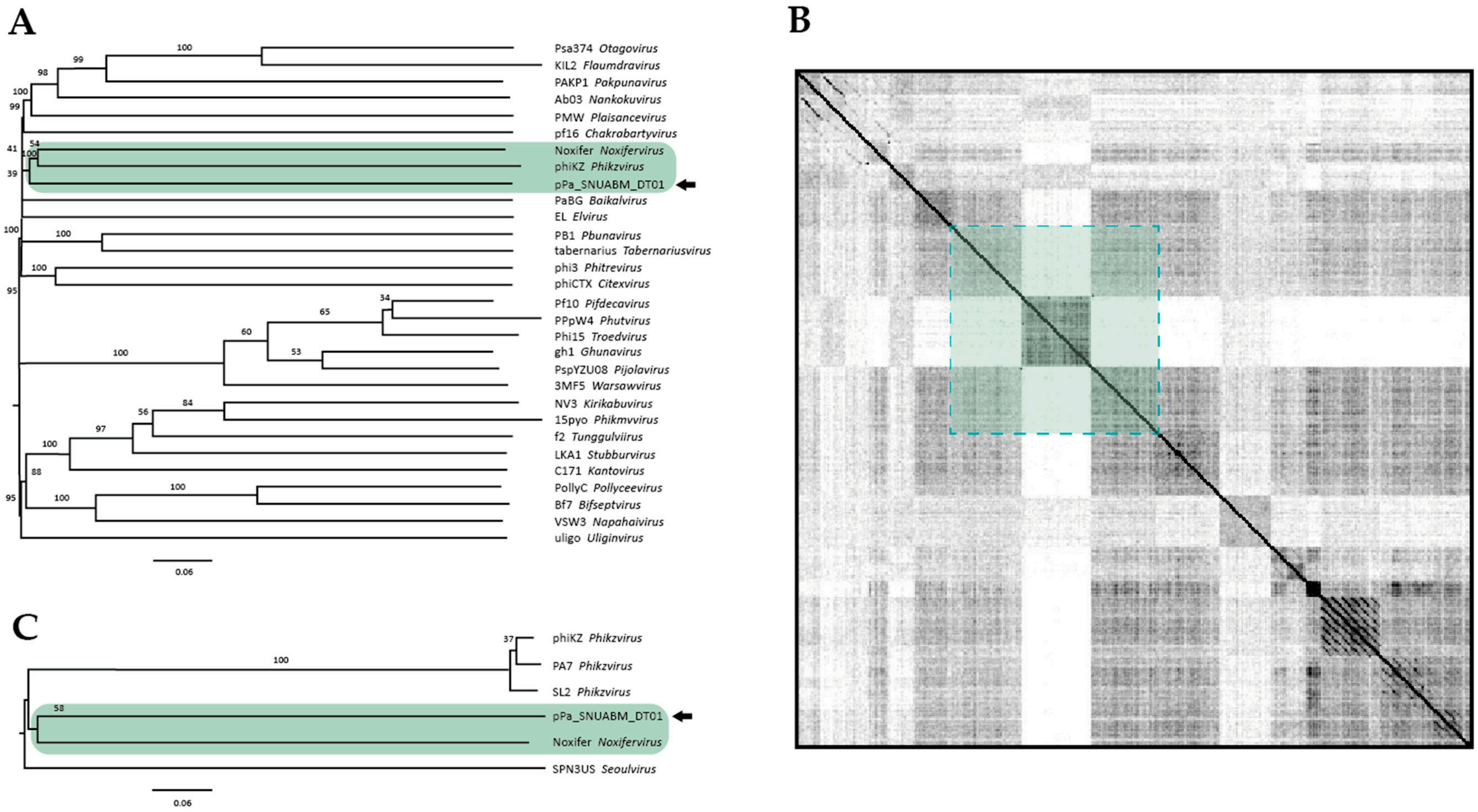

Green box; the cluster including pPa_SNUABM_DT01. (**A**) Phylogenetic tree constructed using VICTOR with genome sequences of genera belonging to *Myoviridae*. (**B**) Dot plot constructed with concatenated genome sequences used in (A) phylogeny. (**C**) Phylogenetic tree constructed using VICTOR with genome sequences belonging to *Noxifervirus* and *Phikzvirus*.
